# PARP1 as a Marker of an Aggressive Clinical Phenotype in Cutaneous Melanoma—A Clinical and an In Vitro Study

**DOI:** 10.3390/cells10020286

**Published:** 2021-01-31

**Authors:** Piotr Kupczyk, Aleksandra Simiczyjew, Jakub Marczuk, Ewelina Dratkiewicz, Artur Beberok, Jakub Rok, Malgorzata Pieniazek, Przemyslaw Biecek, Dmitry Nevozhay, Bartosz Slowikowski, Grzegorz Chodaczek, Dorota Wrzesniok, Dorota Nowak, Piotr Donizy

**Affiliations:** 1 Department of Pathomorphology, Wroclaw Medical University, 50-368 Wroclaw, Poland; piotr.kupczyk@umed.wroc.pl; 2 Department of Cell Pathology, Faculty of Biotechnology, University of Wroclaw, 50-383 Wroclaw, Poland; aleksandra.simiczyjew@uwr.edu.pl (A.S.); ewelina.dratkiewicz@uwr.edu.pl (E.D.); dorota.nowak@uwr.edu.pl (D.N.); 3 Department of Dermatology, Research and Development Center, Regional Specialized Hospital, 51-124 Wroclaw, Poland; jamarczuk93@gmail.com; 4 Department of Pharmaceutical Chemistry, Faculty of Pharmaceutical Sciences in Sosnowiec, Medical University of Silesia, 41-200 Sosnowiec, Poland; abeberok@sum.edu.pl (A.B.); jrok@sum.edu.pl (J.R.); dwrzesniok@sum.edu.pl (D.W.); 5 Department of Clinical Oncology, Tadeusz Koszarowski Regional Oncology Centre, 45-061 Opole, Poland; pieniadzgosia@interia.pl; 6 Faculty of Mathemathics and Information Science, Warsaw University of Technology, 00-662 Warsaw, Poland; przemyslaw.biecek@gmail.com; 7 Department of Imaging Physics, The University of Texas MD Anderson Cancer Center, Houston, TX 77030, USA; dnevozhay@mdanderson.org; 8 School of Biomedicine, Far Eastern Federal University, 690950 Vladivostok, Russia; 9 Department of Biochemistry and Molecular Biology, Poznan University of Medical Sciences, 60-781 Poznan, Poland; slowikowski.bartek@gmail.com; 10 Laboratory of Bioimaging, Łukasiewicz Research Network—PORT Polish Center for Technology Development, 54-066 Wroclaw, Poland; grzegorz.chodaczek@port.lukasiewicz.gov.pl; 11 Department of Pathomorphology and Oncological Cytology, Wroclaw Medical University, 50-556 Wroclaw, Poland

**Keywords:** PARP1, cutaneous melanoma, melanoma cell lines, immunohistochemistry

## Abstract

(1) Background: Poly(ADP-ribose) polymerase 1) (PARP1) is a pleiotropic enzyme involved in several cellular processes, e.g., DNA damage repair, regulation of mitosis, and immune response. Little is known about the role of PARP1 in melanoma development and progression. We aimed to investigate the prognostic significance of PARP1 expression in cutaneous melanoma through evaluation of mRNA and protein levels of PARP1 in normal melanocytes and melanoma cell lines, as well as in patients’ tissue material from surgical resections. (2) Methods: An in vitro model was based on two types of normal human melanocytes (HEMn-DP and HEMn-LP) and four melanoma cell lines (A375, WM1341D, Hs294T, and WM9). *PARP1* mRNA gene expression was estimated using real-time polymerase chain reaction (RT-PCR), whereas the protein level of PARP1 was evaluated by fluorescence confocal microscopy and then confirmed by Western Blotting analysis. The expression of PARP1 was also assessed by immunohistochemistry in formalin-fixed paraffin-embedded tissues of 128 primary cutaneous melanoma patients and correlated with follow-up and clinicopathologic features. (3) Results: The in vitro study showed that melanoma cells exhibited significantly higher PARP1 expression at mRNA and protein levels than normal melanocytes. High PARP1 expression was also associated with the invasiveness of tumor cells. Elevated nuclear PARP1 expression in patients without nodal metastases strongly correlated with significantly shorter disease-free survival (*p* = 0.0015) and revealed a trend with shorter cancer-specific overall survival (*p* = 0.05). High PARP1 immunoreactivity in the lymph node-negative group of patients was significantly associated with higher Breslow tumor thickness, presence of ulceration, and a higher mitotic index (*p* = 0.0016, *p* = 0.023, and *p* < 0.001, respectively). In patients with nodal metastases, high PARP1 expression significantly correlated with the presence of microsatellitosis (*p* = 0.034), but we did not confirm the prognostic significance of PARP1 expression in these patients. In the entire analyzed group of patients (with and without nodal metastases at the time of diagnosis), PARP1 expression was associated with a high mitotic index (*p* = 0.001) and the presence of ulceration (*p* = 0.036). Moreover, in patients with elevated PARP1 expression, melanoma was more frequently located in the skin of the head and neck region (*p* = 0.015). In multivariate analysis, high PARP1 expression was an independent unfavorable prognosticator in lymph node-negative cutaneous melanoma patients. (4) Conclusions: In vitro molecular biology approaches demonstrated enhanced PARP1 expression in cutaneous melanoma. These results were confirmed by the immunohistochemical study with clinical parameter analysis, which showed that a high level of PARP1 correlated with unfavorable clinical outcome. These observations raise the potential role of PARP1 inhibitor-based therapy in cutaneous melanoma.

## 1. Introduction

Melanoma represents less than 5% of all cutaneous malignancies, but accounts for the majority of skin cancer deaths [1]. The effectiveness of melanoma treatment is continuously improving; nevertheless, a substantial number of patients are still being diagnosed with tumors in the advanced stage, which cannot be treated exclusively by means of surgical excision, and the adjuvant therapy does not result in a full recovery [1,2].

Over the last few years, the dynamic development of personalized therapy has been observed, which also concerns melanoma patients. The main groups of new drugs applied in the treatment of this type of tumor encompass immunotherapeutic agents (e.g., immune checkpoint inhibitors) and molecularly targeted drugs directed against type B rapid accelerated fibrosarcoma/mitogen activated protein kinases (BRAF/MEK). Current advances in dermato-oncology have led to a five-year survival rate of about 50% in patients with metastatic cutaneous melanoma [3]. However, their effectiveness is limited due to resistance acquisition and/or the appearance of many different mutations in the tumor cells, varying from patient to patient and also within the tumor in the same patient [4]. For this reason, it is necessary to pursue new therapeutic solutions [2,5].

One of the new treatment options could be the application of poly (ADP-ribose) polymerase 1 (PARP1) inhibitors, which are already used in breast and ovarian cancer therapy [6]. This protein belongs to the PARP enzyme family comprising 17 or 18 members (depending on the classification) [7,8,9], which catalyze the reaction of ADP-ribosylation, that is, ADP-ribose transfer from nicotinamide adenine dinucleotide (NAD+) to another target molecule [7,8,9,10,11].

PARP1 is an important enzyme participating in several cellular processes such as DNA damage repair, various types of cell death (parthanatos, apoptosis, necroptosis or autophagy), synthetic lethality, regulation of chromatin structure, DNA methylation, regulation of transcription (and thereby the development of an inflammatory process or cell differentiation), and finally carcinogenesis—mainly by regulating transcription of genes encoding proteins involved in cancer progression [11,12,13,14,15,16,17,18,19,20,21,22].

The role of PARP1 in carcinogenesis depends on various, sometimes contradictory, mechanisms. PARP1 may modify the transcription of various tumor suppressors with the forefront of p53. On the other hand, increased activity of PARP1 may initiate the T-cell factor/lymphoid enhancer-binding factor, a protein family of transcription factors, which under normal conditions controls organ development, while its appearance in cancers promotes its progression (e.g., colon cancer) [18,23]. PARP1 was found to be a cofactor for adenovirus E2 promoter-binding protein (E2F-1), a transcription factor with dual oncogene or tumor suppressor functions and abilities to control the balance between proliferation and cell apoptosis. E2F-1 directly interacts with PARP1 and increases its promoter activity as well as associating signaling molecules independently on the PARP1 enzymatic activity or DNA binding. Thus, the PARP1-E2F-1 complex may exhibit protooncogene functions, which was demonstrated in prostate cancer cells. Furthermore, PARP1 was engaged in the promotion of oncogenic fusion genes, e.g., transmembrane protease serine 2 (TMPRSS2) - erythroblast transformation-specific related gene (ERG) (TMPRSS2-ERG) in prostate cancer or Ewing’s sarcoma breakpoint region (EWS) - Friend leukemia integration 1 transcription factor (FLI1) (EWS-FLI1) and EWS-ERG in Ewing’s sarcoma [24,25].

PARP1 also plays a role as a multifactorial regulator of epithelial–mesenchymal transition (EMT), regarded as a key mechanism enabling metastasis formation. Inhibition of PARP1 activity leads to a reduction in Snail1 and vimentin expression and upregulation of the E-cadherin level, which significantly abolished metastasis [26]. Furthermore, PARP1 regulates the cellular response to hypoxia, as well as angiogenesis, by enhancement of the expression and activity of HIF1α, HIF2α, and VEGF-A, which improves blood supply to the cells (including the malignant ones) [18,24]. PARP1 also participates in hormone-dependent tumor pathogenesis. In breast cancer, it is considered a positive regulator of the level of estrogen α and β, while in prostate cancer, it activates the androgen receptor [18].

PARP1 takes part in various mechanisms leading to the development of resistance to conventional cancer therapy, including radiotherapy, chemotherapy, and hormonal therapy. PARP1 activates the MAPK and mTOR pathways and thus induces authophagy, resulting in the radioresistance of nasopharyngeal cancer [27]. It also PARylates the estrogen receptor α, which is associated with resistance to tamoxifen in breast cancer patients [18,28,29]. In both cases, application of PARP1 inhibitors led to the sensitisation to applied treatment [27,28,29].

The aim of the study was to explore the possible involvement of PARP1 in melanoma development and progression. To realize this purpose, we performed in vitro cell culture studies using two normal melanocyte cell cultures (HEMn-LP and HEMn-DP) and four malignant melanoma cell lines exhibiting various level of invasiveness (two skin-derived cell lines: A375 and WM1341D, and two metastatic lymph node-derived cell lines: Hs294T and WM9). PARP1 gene and protein expression were analyzed using RT-PCR as well as Western Blotting and confocal microscopy. To confirm the obtained results, we performed the immunohistochemical evaluation of PARP1 protein expression in tumor cells of 128 skin melanoma patients, followed by an analysis of the correlation between PARP1 expression and detailed clinical and histopathological parameters, as well as patient survival.

To the best of our knowledge, this is the first comprehensive in vitro and clinical study of PARP1 expression utilizing a well-defined in vitro model based on two kinds of normal primary melanocytes and four melanoma cell lines and a large cohort of cutaneous melanoma patients with long-term follow-up.

## 2. Materials and Methods

### 2.1. Cell Culture

Human normal epidermal melanocytes, lightly (HEMn-LP) and darkly pigmented (HEMn-DP), were cultured in M-254 growth medium supplemented with human melanocyte growth supplement-2 (HMGS-2) (Cascade Biologics/Gibco, Carlsbad, CA, USA), 10 μg/mL neomycin (Amara, Cracow, Poland), 100 U/mL penicillin, and 0.25 μg/mL amphotericin B (Sigma–Aldrich, St. Louis, MO, USA). All experiments were performed using cells at passage 6. Human melanoma cell lines derived from primary tumors—A375, WM1341D, and from lymph nodes metastases—Hs294T, WM9 (American Type Culture Collection—ATCC, Rockland Immunochemicals, Inc., Limerick, PA, USA) were grown in DMEM (Dulbecco’s modified Eagle’s medium) containing 4.5 g/l glucose and 1.5 g/l NaHCO_3_ supplemented with 10% FBS, 2 mM L-glutamine, and antibiotics (100 U/mL penicillin, 0.1 mg/mL streptomycin, 0.25 µg/mL amphotericin B (Sigma–Aldrich)). All cells were cultured in 25 cm^2^ tissue culture flasks (Sarstedt Inc., Nümbrecht, Germany) and were maintained at 37 °C in 5% CO_2_/95% humidified air and were passaged twice a week using 0.25% trypsin/0.05% EDTA solution (Institute of Immunology and Experimental Therapy, Polish Academy of Sciences—IIET, PAS, Wroclaw, Poland).

### 2.2. Confocal Microscopy and Image Analysis

The microscope imaging was performed using a Zeiss Cell Observer SD spinning disk confocal microscope (Carl Zeiss, Jena, Germany) equipped with a dry 20× objective. Laser lines used for excitation were 405 nm (DAPI—nuclei), 488 nm (DyeLight488—PARP1), and 568 nm (Alexa Fluor555—F-actin), and constant camera settings and exposure time (300 ms) were applied. The imaging was performed in two independent cultures, and five randomly selected areas from culture plate wells were further analyzed to evaluate the PARP1 level. ZEN software (Carl Zeiss) was used for the generation of a series of z-stack images. Further steps of fluorescence intensity (FI) analysis were performed using Fiji-ImageJ software (National Institutes of Health, Bethesda, MD, USA). First, the Subtract Background and Filters > Median algorithms were applied to reduce the background noise. To measure the cellular PARP1 level, cell bodies were identified based on the F-actin staining, which was used to extract cellular regions of interest (ROIs). The F-actin channel was thresholded with the Huang algorithm to detect cellular ROIs, and binary images were obtained showing cell bodies. Overlapping cell objects were separated using the Erode and Watershed algorithms. The Analyze particles function was utilized to detect cellular ROIs in all ten images. Finally, identified ROIs were transferred onto the PARP1 channel and PARP1 FI per cell was measured using the Analyze particles function again.

### 2.3. Cell Lysate Preparation

Melanocytes and melanoma cells (A375, WM9, Hs294T, and WM1341D cell lines) were seeded onto 60 mm Petri culture dishes and allowed to reach the minimal 70% confluence. Then, cells were placed on ice, washed with PBS, lysed in radioimmunoprecipitation assay (RIPA) lysis buffer (50 mM TRIS-HCl pH 7.0, 150 mM NaCl, 1% NP-40, 0.1% SDS, 1% sodium deoxycholate) containing phosphatase and protease inhibitors (Sigma-Aldrich) and frozen at −80 °C. After thawing, the lysates were centrifuged at 4 °C for 10 min at 12,000× *g*. Supernatants were transferred into fresh tubes, and the protein content was measured using the standard Bradford method [30].

### 2.4. Western Blotting Analysis

Samples of an identical amount of protein (5 μg) were separated using SDS-PAGE electrophoresis according to Laemmli [31] and were then transferred to nitrocellulose sheets, according to the Towbin et al. protocol [31,32]. The quality of transfer was determined with Ponceau S staining. Then, the membranes were blocked with 5% non-fat milk in Tris-Buffered Saline with Tween 20 (TBST) for 1 h at RT and incubated overnight at 4 °C with primary mouse antibodies directed against PARP1 (SC-74470; dilution 1:500; clone B-10, Santa Cruz Biotechnology, Santa Cruz, CA, USA) and glyceraldehyde 3-phosphate dehydrogenase (GAPDH) (SC-365062, Santa Cruz Biotechnology, Santa Cruz, CA, USA), which was used as an internal loading control. This was followed by a 1 h incubation with secondary antibodies conjugated with horseradish peroxidase directed against primary mouse antibodies (Cell Signaling, Danvers, MA, USA). Immunoblots were developed using the Clarity Western ECL Substrate (Bio-Rad, Hercules, CA, USA), scanned with ChemiDoc (Bio-Rad, USA) and were analyzed with ImageLab software (ver. 6.0, Bio-Rad, USA). The experiment was performed in triplicate (three biological repetitions, each consisting of two–three technical replicates).

### 2.5. RNA Isolation and Reverse Transcription

The total RNA from normal melanocytes and malignant melanoma cells was isolated using a combined protocol based on Trizol (Ambion Inc., Austin, TX, USA) reagent and the GENEMatrix Universal RNA Purification Kit (EurX—Molecular Biology Products, Gdansk, Poland) according to a previously described protocol with minor modifications [33]. Briefly, confluent cells were washed with PBS, scraped in Trizol reagent, and frozen at −80 °C. Then, cells were removed from the freezer, transferred to a thermoblock (Eppendorf, Hamburg, Germany), and incubated for 30 min at 4 °C at 1400 rpm. Following the addition of chloroform (POCH, Gliwice, Poland) and centrifugation (12,500× *g*, 15 min, 4 °C), the upper clear RNA-containing phase was carefully removed and mixed with isopropanol (POCH, Gliwice, Poland). After that, the whole volume was transferred to a new tube with a homogenization membrane and centrifuged (11,000× *g*, 3 min, 4 °C ) to eliminate cellular debris and genomic DNA (gDNA). The flow-through was placed on the silicone-binding membrane and centrifuged (11,000× *g*, 1 min, 4 °C). Next, the flow-through was discarded, and after the addition of DN1 wash buffer, the samples were centrifuged. To remove any residual contamination of gDNA, an additional step consisting of treatment with DNase I Kit (EurX—Molecular Biology Products, Gdansk, Poland) was performed. Then, samples were incubated for 15 min at RT, followed by a washing step (2× RBW wash buffer) and centrifugation (as described above). All silicone membranes were removed and transferred to a new collection tube where RNA/DNA-free water (EurX—Molecular Biology Products, Gdansk, Poland) was added. The centrifugation step (12,500× *g*, 2 min) allowed us to obtain total RNA-rich samples for which the quantity and quality were estimated using Implen NanoPhotometer (Implen Inc., Munich, Germany). Only RNA samples with an absorbance 260/280 ratio ranging between 1.7–2.1 were used for the synthesis of 0.5 μg of cDNA using the smaRT First Strand cDNA Synthesis Kit (EurX—Molecular Biology Products, Gdansk, Poland) according to the manufacturer’s protocol.

### 2.6. Real-Time PCR Gene Expression Analysis

Following cDNA synthesis, samples were diluted with RNA-free water. For gene expression analysis, 1 μL of cDNA (10 ng) per reaction was used in a total volume of 10 μL. The reaction mix (per well) also included 5 μL of UPL ProbeMaster (Roche, Risch-Rotkreuz, Switzerland), 0.5 μM of forward and reverse primers (Genomed, Warsaw, Poland), and 0.2 μM of Universal Probe Library (UPL, Roche) hydrolysis probes for target gene *PARP1* and three housekeeping genes *POLR2A*, *PPIA*, and *GAPDH*. All primers and probes were designed utilizing ProbeFinder Software (Roche), and their respective sequences as well as UPL probe numbers are listed in the table (Table 1). The real-time PCR was performed using a LightCycler 480 II (Roche Molecular Systems Inc., Indianapolis, IN, USA) instrument with following conditions: pre-incubation at 95 °C for 10 min, 50 cycles of amplification: 15 s at 95 °C for denaturation, 30 s at 60 °C for annealing, and 10 s at 72 °C for elongation, followed by cooling at 40 °C for 10 s. The measurements of the target gene were normalized to the geometric average of the values of all three housekeeping genes, and relative *PARP1* gene expression was presented using the 2^−ΔΔCT^ method [34]. All gene expression analyses were performed in triplicate in the three independent experiments.

### 2.7. Immunohistochemistry

Immunohistochemical evaluation was performed using mouse monoclonal anti-human PARP1 antibody (SC-74470; dilution 1:50; clone B-10, Santa Cruz Biotechnology) on 4-μm-thick paraffin sections mounted on silanized slides (code number S3003; Dako Denmark A/S, Glostrup, Denmark). The sections were then deparaffinized, rehydrated, and subjected to heat-induced epitope unmasking. The pT Link module was applied for this purpose using EnVision™ Target Retrieval Solution (20–40 min incubation at 97 °C). An immunohistochemical test was performed utilizing Autostainer Link and EnVision™ FLEX/HRP (SM802; Dako). Stained human placental tissue was used as a positive control. Negative controls were processed with FLEX Rabbit Negative Control, Ready-to-Use (Agilent DAKO) in place of the primary antibody.

Scoring of PARP1 immunostains was performed using the H-score [(percentage at 1+) × 1 + (percentage at 2+) × 2 + (percentage at 3+) × 3], which integrates the intensity and percentage of positive cells into a combined score. The H-score of 280 was used as a cut-off value for high (H-score > 280) and low PARP1 (H-score ≤ 280) expression [35].

### 2.8. Patients

Our study group was composed of 128 cutaneous melanoma patients treated at the Regional Oncology Centre in Opole, Poland, diagnosed in 2005–2010. Patients were enrolled in this study based on the availability of their medical documentation and tissue material, which included paraffin blocks and histopathology slides. Comprehensive clinical data were retrieved from the archival medical records, and data concerning the diagnostic and therapeutic procedures used were sourced from the cancer outpatient clinic at the Regional Oncology Centre in Opole, Poland.

This retrospective study was reviewed and approved by the ethics committee of the Wroclaw Medical University, Wroclaw, Poland (No 277/2020). The patients did not personally participate in the study, and the results of these investigations did not have any influence on the original treatment of patients because it had already been finished. All investigations were performed in accordance with the Declaration of Helsinki.

Records were reviewed for clinical and pathological data such as age, gender, location, presence or absence of nodal and distant metastases, information concerning disease recurrence, and sentinel lymph node biopsy (SLNB) procedures (Table 2).

Histopathologic parameters (Breslow thickness, Clark level, histological type, mitotic rate (number of mitotic figures per 1 mm^2^), presence of ulceration, lymphangioinvasion, microsatellitosis, intensity of tumor-infiltrating lymphocytes (TILs), and microscopic evidence of regression, were evaluated based on hematoxylin and eosin (H&E) staining from sections of archival formalin-fixed, paraffin-embedded tumor specimens (Table 3).

### 2.9. Statistical Analysis

For in vitro analyses, real-time PCR and Western Blotting experiments were performed in triplicate, whereas confocal microscopy was performed in duplicate, and obtained data were analyzed using STATISTICA 13.1 (Dell) software. ANOVA and subsequent pair-wise post-hoc comparisons were performed using the Tukey HSD test where applicable. A *p* value below 0.05 was considered significant for all comparisons, and *p* values presented on the bar graphs were as follows: *p* < 0.05–0.01 (*); 0.01–0.001 (**); 0.001–0.0001 (***); and *p* < 0.0001 (****). 

Statistical analysis of parameters from the histopathologic evaluation of patient-derived tumor tissues was performed using R language [R Core Team. R: A language and environment for statistical computing. R Foundation for Statistical Computing, Vienna, Austria, https://www.r-project.org/ (2019, accessed on 12 March 2020)] and the survminer tool [36]. For the purposes of correlation analysis, we assumed a dichotomous division of PARP1 expression into low and high corresponding to a semiquantitative H-score of ≤280 and >280, respectively. Kaplan–Meier curves and the log-rank test were used to determine the cancer-specific overall survival (CSOS) and disease-free survival (DFS); all analyses were carried out using the survival package for R [R Core Team. R: A language and environment for statistical computing. R Foundation for Statistical Computing, Vienna, Austria, https://www.r-project.org/ (2019, accessed on 12 March 2020); [36]. In order to determine the correlations between the PARP1 expression and continuous variables, the Wilcoxon two-sample test was used. The correlations between PARP1 expression and binary variables were determined using Fisher’s exact test, while the correlations with other categorical variables were determined using the chi-square test.

The graphs were prepared using GraphPad Prism 7 (GraphPad Software Inc., La Jolla, CA, USA), while figures by LibreOffice 5.0 Software (The Document Foundation, Berlin, Germany).

## 3. Results

### 3.1. PARP1 in Normal Melanocyte and Malignant Melanoma Cell Lines

**Figure 1:**
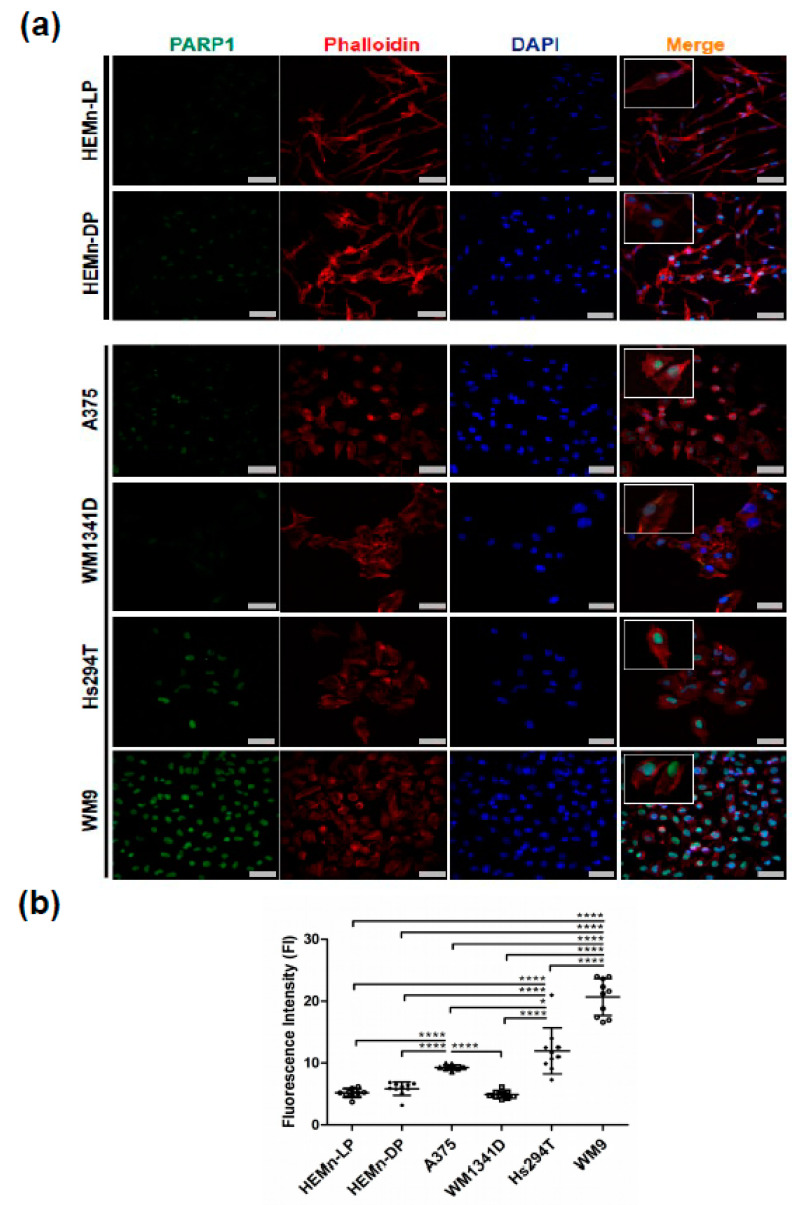
PARP1 localization and fluorescence intensity analysis in normal melanocytes and selected melanoma cell lines. Representative images of normal melanocytes (HEMn-LP and HEMn-DP) and melanoma cell lines derived from the primary tumor (A375 and WM1341D) and lymph node metastases (Hs294T and WM9) stained for PARP1 (green), phalloidin for F-actin (red), and DAPI for cell nuclei (blue) (**a**). Scale bar—20 μm. The magnification per cell in the merged images was used for better representation of nuclear differences in PARP1 expression per cell between normal melanocytes and melanoma cell lines. Fluorescence intensity of the PARP1 signal presented as a mean (N = 10) ± standard deviation (SD) (**b**). Analysis was performed in Fiji-ImageJ. The significance level was set at *p* = 0.05–0.01 (*), *p* ≤ 0.0001 (****).

In two normal human melanocyte cell cultures (HEMn-LP and HEMn-DP) and four melanoma cell lines (A375, WM1341D, Hs294T, and WM9), PARP1 protein localization was visualized using confocal microscopy. It was present in the nuclear area, which is clearly visible on the overlay with DAPI staining (Figure 1a). F-actin was stained to visualize the cell shape in the examined cells. We observed that the signal from PARP1 in normal melanocytes and the WM1341D melanoma cell line was weaker than the same signal in A375, Hs294T, and WM9 melanoma cells.

We decided to perform a quantitative analysis of the fluorescence intensity (FI) of the PARP1 signal. The obtained results are shown in Figure 1b. The FI was the highest in WM9 and Hs294T melanoma cell lines, which represent metastatic melanoma. Its elevated levels were observed in the A375 cell line, while the signal in WM1341D was comparable to the FI in normal melanocytes.

**Figure 2:**
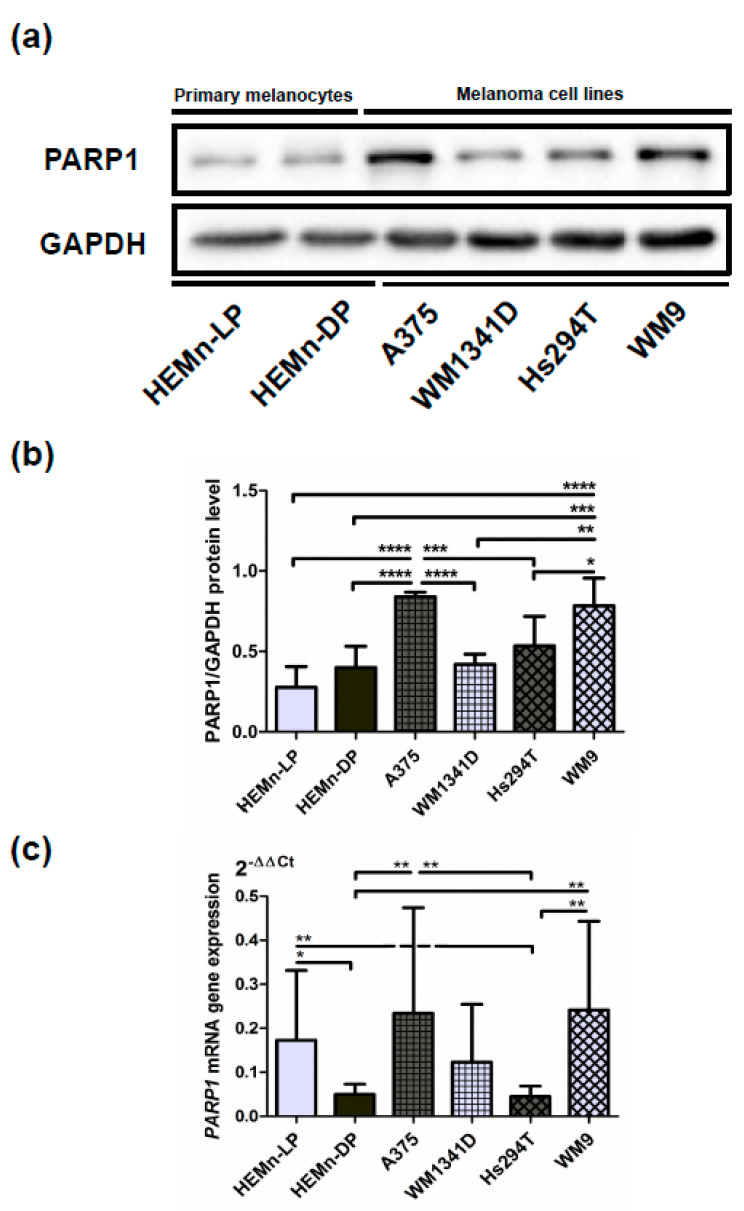
Expression of PARP1 at protein and gene *PARP1* mRNA levels. Representative immunoblots showing PARP1 and GAPDH levels in cellular extracts of primary normal melanocytes (HEMn-LP and HEMn-DP) and melanoma cell lines derived from the primary tumor (A375 and WM1341D) and lymph node metastases (Hs294T and WM9) are presented (**a**). Results of densitometric analysis of the PARP1 level (adjusted to GAPDH) are shown as a mean (N = 3) ± SD (**b**). *PARP1* gene expression analysis at the mRNA level was performed using real-time PCR in the examined cells in triplicate. Results are expressed as a mean (relative gene expression compared to three housekeeping genes—*POLR2A, PPIA, G3PDH*) (N = 3) ± SD of three independent experiments (**c**). The significance level was set at *p* = 0.05–0.01 (*), *p* = 0.01–0.001 (**), *p* = 0.001–0.0001 (***), *p* ≤ 0.0001 (****).

To confirm these results, we performed Western blotting analysis, which showed similar results. Again, the level of PARP1 was higher in WM9, Hs294T, and A375 cells compared with WM1341D cells and normal melanocytes (Figure 2a,b). All analyzed immunoblots are included in Supplemental Figure S1, while Supplemental Figure S2 shows the specificity of the anti-PARP1 antibody used for Western Blotting analysis.

Moreover, the PARP1 mRNA level was evaluated with the RT-PCR method. Here, we also observed a high transcript level in WM9 and A375 cells. WM1341D and low-pigmented melanocytes exhibited intermediate expression, whereas Hs294T and high-pigmented melanocytes exhibited the lowest expression (Figure 2c).

### 3.2. Expression of PARP1 in Cutaneous Melanoma Patients

**Figure 3:**
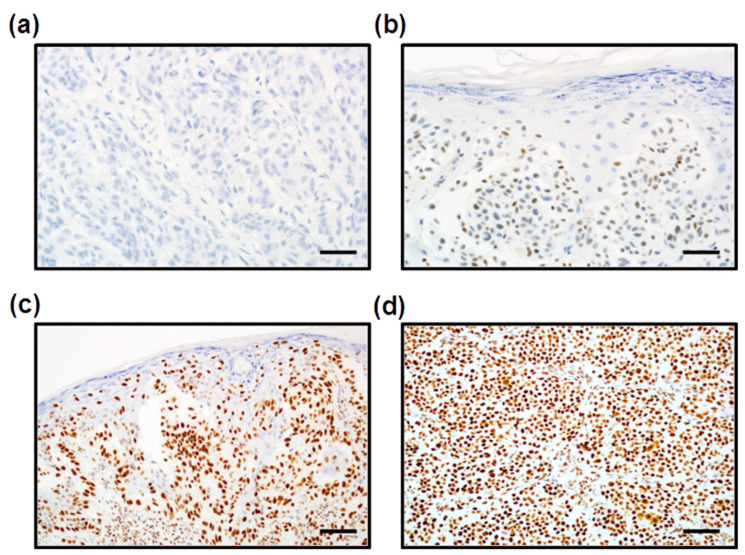
Immunohistochemical analysis of PARP1 expression in cutaneous melanoma patients. Lack of PARP1 immunoreactivity in melanoma cells ((**a**), 400×). Low nuclear expression of PARP1 in melanoma cells ((**b**), 400×). High PARP1 expression localized in the nuclei of melanoma cells ((**c**), 200×; (**d**), 200×). Scale bars for 200× and 400× are 200 μm and 100 μm, respectively.

PARP1 expression was evaluated by immunohistochemistry performed on 128 whole tumor slides from archival formalin-fixed, paraffin-embedded specimens. PARP1 immunoreactivity was measured with the H-score method. PARP1 H-scores ranged from 0 to 300, and the mean H-score value was 229.88 (±67.68), median: 245. In all positive cases, we observed nuclear PARP1 localization (Figure 3). For the statistical analysis, we divided the study group into two subgroups: [1] low PARP1 expression (defined as an H-score ≤ 280) and [2] high PARP1 expression (defined as an H-score >280). Low PARP1 immunoreactivity was observed in 98 patients (76.6%), whereas high PARP1 expression was observed in 30 patients (23.4%).

### 3.3. Correlation between PARP1 Expression and Clinicopathologic Parameters of Primary Tumors

Enhanced PARP1 immunoreactivity in melanoma cells in the whole group of patients (with and without lymph node metastases) was significantly correlated with a high mitotic index and the presence of ulceration (*p* = 0.001 and *p* = 0.036, respectively). Primary tumors localized particularly in the head and neck region were characterized by high PARP1 immunoreactivity (*p* = 0.015) (Table 2).

In patients without lymph node metastases, high PARP1 expression was significantly associated with thick (according to the Breslow scale), ulcerated, and highly mitogenic primary tumors (*p* = 0.0016, *p* = 0.023, and *p* < 0.001) (Table 3), whereas in patients with nodal metastases, upregulation of PARP1 correlated with the presence of microsatellitosis (*p* = 0.034) (Supplemental Table S1).

Statistical analysis did not reveal any other significant associations between PARP1 expression and clinicopathologic parameters in lymph node-negative and lymph node-positive patients (Table 4 and Table 5).

### 3.4. Impact of PARP1 Expression on Long-Term Survival in Cutaneous Melanoma Patients

**Figure 4:**
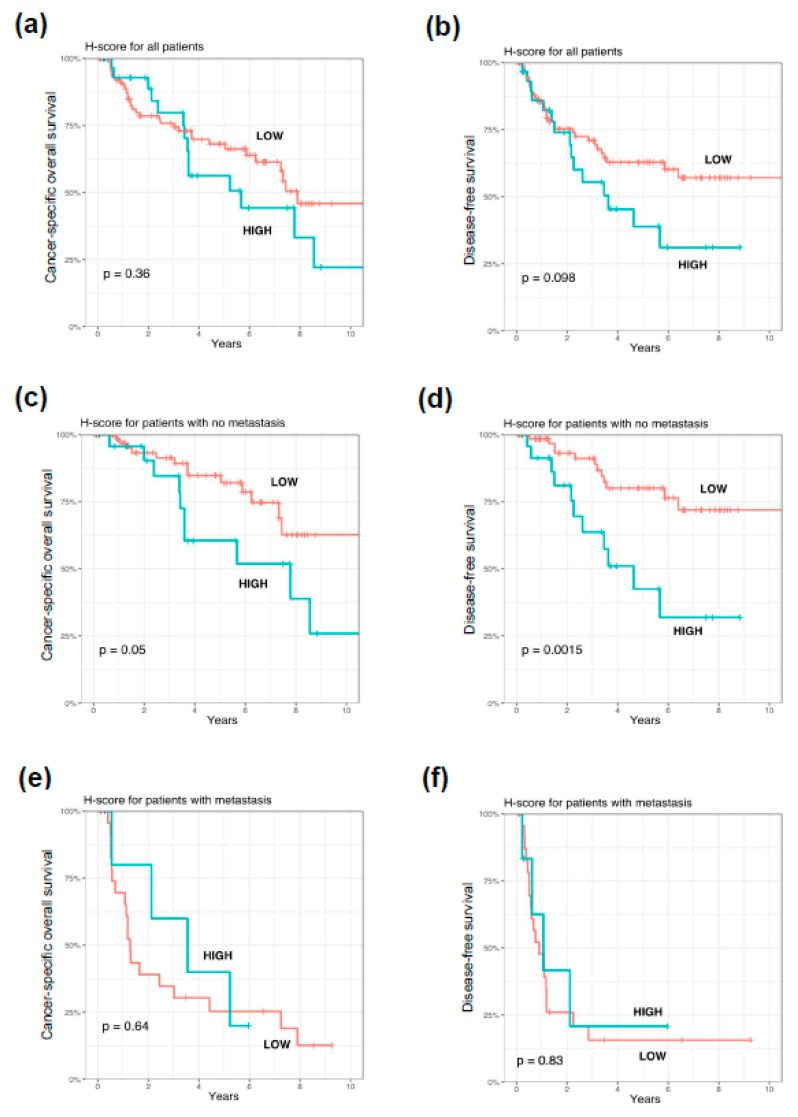
Kaplan–Meier analysis of the prognostic significance of PARP1 expression in cutaneous melanoma patients. PARP1 overexpression had no prognostic significance in the entire study group of cutaneous melanoma patients (**a**,**b**). In the lymph node-negative subgroup of cutaneous melanoma patients, increased PARP1 immunoreactivity corresponded with shorter cancer-specific overall survival (**c**) and significantly correlated with reduced disease-free survival (**d**). Prognostic significance of PARP1 immunoreactivity in lymph node-positive melanoma patients was not observed (**e**,**f**). *p* levels of the log-rank test.

High PARP1 expression in tumor cells was significantly correlated with shorter cancer-specific overall survival (*p* = 0.015) in lymph node-negative patients (Figure 4). We observed a trend between enhanced PARP1 expression and shorter disease-free survival (*p* = 0.05) in patients without nodal metastases. PARP1 immunoreactivity had no impact on survival in the whole group and the subgroup of lymph node-positive cutaneous melanoma patients (Figure 4).

**Figure 5:**
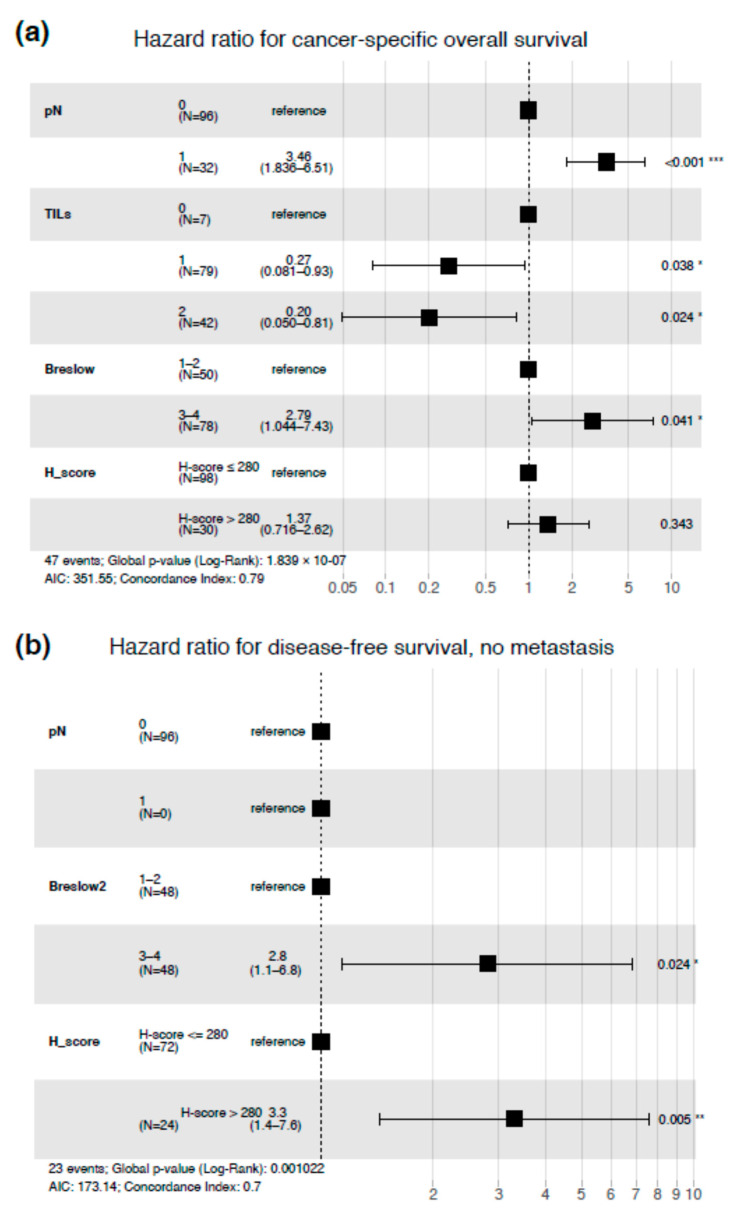
Multivariable regression model for cancer-specific overall survival in the entire study group of cutaneous melanoma patients (**a**) and multivariable regression model for disease-free survival in lymph node-negative melanoma patients. TILs = tumor-infiltrating lymphocytes (**b**). Stars corresponds to statistical significance, * stands for *p*-value < 0.05, ** for *p*-value < 0.01, *** for *p*-value < 0.001.

The multivariable Cox regression model was created to test whether PARP1 expression may be used as an independent prognostic factor. After adjustment for Breslow thickness, high PARP1 expression was associated with shorter DFS (HR = 3.3, *p* = 0.005) in lymph node-negative patients (Figure 5). In the whole cohort of patients, only the Breslow thickness, nodal status, and tumor-infiltrating lymphocytes were independent prognosticators in the context of CSOS (Figure 5).

## 4. Discussion

Our in vitro studies showed that melanoma cells exhibited significantly higher PARP1 expression compared with normal melanocytes. Moreover, the high PARP1 level was associated with increased invasiveness of tumor cells. Experiments performed on patients’ surgical resection specimens demonstrated that PARP1 overexpression significantly correlated with shorter disease-free survival in patients without nodal metastases. Up-regulation of PARP1 in melanoma cells in the whole cohort of patients had strict correlations with unfavorable histopathologic parameters such as greater Breslow thickness, presence of ulceration, and high mitotic activity.

In our previous studies, we demonstrated that the WM1341D cell line exhibits lower invasive abilities than A375, WM9, and Hs294T cell lines [37,38]. Compared to other cells, WM1341D formed a lower number of invadopodia (adhesive structures with proteolytic activity), which are utilized by cancer cells to digest the extracellular matrix and invade tissue. The highest number of this type of protrusion was formed by WM9 cells. These cells were also the most effective in digesting fluorescently-labeled gelatin in a test that allows us to determine the proteolytic capacity of the examined cells. These abilities have a direct effect on cell invasive potential. The WM9 cells were not only able to digest the largest area of gelatin of all tested lines, but also the highest percentage of cells exhibited proteolytic activity. The smallest number of digesting cells as well as the lowest digestion area were observed in WM1341D cells. A375 and Hs294T cells digested to a greater extent than WM1341D [37,38]. Our current results indicate that the invasiveness of the examined melanoma cells correlated positively with the level of PARP1 in these cells. The least amount of PARP1 was observed in the WM1341D cell line, which had the lowest invasive potential among all analyzed cell lines and displayed PARP1 mRNA and protein levels closest to the non-malignant melanocytes.

So far, in vitro and clinical studies are in line with our observations and seem to indicate a significant role of PARP1 in melanoma progression and the development of metastases [35]. Staibano et al., using immunohistochemical methods and Western Blotting, observed that elevated PARP1 expression in patients with melanoma in the head and neck region was associated with transition from the radial to the vertical growth phase and was positively correlated with the tumor thickness in the vertical growth phase (but not the radial one) [39]. Moreover, proteomic analysis of samples derived from patients with stage III skin melanoma (according to the 7th edition of AJCC staging) revealed that elevated PARP1 expression might be one of the markers discriminating patients with better prognosis from those with the worse one [40]. In a similar study, Davis et al. also observed a significant correlation between elevated PARP1 expression and the presence of ulceration, which is a parameter that negatively affects prognosis [41].

Amiri et al., using an in vitro model based on the Hs294T melanoma cell line and cultures of normal melanocytes, showed that the enzymatic activity of PARP1 was increased in malignant cells when compared to normal melanocytes, which resulted in PARylation of various proteins (and of PARP1 itself) with secondary dissociation of NF-κB from PARP1, binding of NF-κB to the CXCL1 gene promoter, and an increase in its transcription, which may indicate a potential role of PARP1 in the regulation of the immune response [42,43]. Our study did not show any significant correlation between PARP1 immunoreactivity and tumor-infiltrating lymphocyte (TIL) grade within the tumor. However, the mechanism of TIL infiltration in a PARP1-dependent manner might be more complex. The mouse experimental breast cancer model showed that coordinated double co-silencing of PARP1-/- and PARP2-/- is sufficient for complete impairing of TILs, which was not observed in the tumors with functionally activated PARP1 and PARP2 proteins [44].

Experimental studies showed promising results of PARP1 inhibitor (PARP-i) efficacy in melanoma treatment. It was demonstrated in human (G361) and murine (B16-F10) melanoma cell lines that PARP-i reduced cells’ ability to form metastases by downregulation of vascular mimicry—a phenomenon characteristic of an aggressive phenotype of tumor cells capable of differentiating into endothelial-like cells and supporting cancer angiogenesis [26,45]. In another study, application of veliparib (ABT-888, PARP1 inhibitor) resulted in an increased apoptosis level, decreased migration, and invasiveness of the A375 melanoma cell line. Furthermore, these effects were particularly evident in the A375R cell line, which exhibited resistance to dabrafenib, a mutated BRAF inhibitor, which opens up a new perspective for PARP-i use in the case of resistant melanoma treatment [46]. Raineri et al. showed that A375 cells treated with PARP1-i, AZD2461 in combination with onconase, a ribonuclease enzyme with strong antitumor activity in a number of cancers, inhibited the PARP1-NF-κB pathway, which resulted in reduced cell colony formation, migration, and invasion as well as elevated induction of apoptosis [47,48]. Moreover, another PARP-i, olaparib, although used as a monotherapy, does not exert any significantly therapeutic effect in uveal melanoma patients; however, when combined with dacarbazine, it increased its effectiveness [49]. In another study utilizing uveal melanoma cell lines, it was reported that inhibition of PARP1 led to retardation or almost complete inhibition of tumor development [50].

PARP1 is an important mitotic-related protein that decreases the PARP1 protein level promoted cell cycle arrest at prophase [35,51]. In acute myeloid leukemia, inhibition of PARP1 induced neoplastic cell apoptosis and arrested cell cycle in G2/M phase [52]. In line with the cited molecular studies, we observed a strong correlation between PARP1 overexpression and high mitotic activity. Moreover, in mucosal melanoma, upregulation of PARP1 in neoplastic cells was also associated with an enhanced mitotic index, which confirms a significant role of PARP1 in the regulation of mitosis in melanomagenesis. Due to the significant correlation between high PARP1 expression and enhanced mitotic activity, potential inhibition of PAPR1 in cutaneous melanoma patients could be beneficial. Further clinical studies are needed to clarify this therapeutic approach in cutaneous melanoma.

In the current study, we revealed a significant correlation between the high expression of PARP1 and localization of the primary tumor in the head and neck region (vs. trunk and extremities). As this region is the most exposed to ultraviolet (UV) radiation, PARP1 overexpression observed in this localization is a possible immunohistochemical indicator of cumulated DNA aberrations caused by UV. Our observations are in line with the results of previous studies. Robu et al. confirmed the induction of PARP1 expression following the application of UV radiation in in vitro and in vivo models, while PARP1 silencing significantly impaired DNA repair processes, where a key role is played by this enzyme. It was also shown that PARP1 catalytic activity is maintained by DNA-binding protein 2 (DDB2), which is an important part of the repair mechanism based on the nucleotide excision repair pathway (NER). Moreover, the DDB2–PARP1 complex was observed in regions with significant UV-induced chromatin damage [53].

## 5. Conclusions

To conclude, in our in vitro model, increased PARP1 expression was associated with enhanced invasiveness of melanoma cell lines. Elevated levels of PARP1 are also an independent negative prognostic marker in lymph node-negative cutaneous melanoma and correlated with aggressive clinical phenotypes in the analyzed cohort of patients. These observations raise the potential role of PARP1 inhibitor-based therapy in cutaneous melanoma. Further in vitro and clinical studies are warranted.

## Figures and Tables

**Table 1:** PARP1 target gene-specific sequences and housekeeping genes (HKGs) and their respective FAM-labeled universal probe library (UPL) probes and gene association numbers for real-time PCR.

Genes	Forward (F) and Reverse (R) Primers Sequences	UPL Probes	Gene Accession Number
	**Target Gene**		
*PARP1*	F: TCTTTGATGTGGAAAGTATGAAGAA R: GGCATCTTCTGAAGGTCGAT	#22	NM_001618.3
	**Housekeeping Genes (HKGs):**		
*POLR2A*	F: TCCGTATTCGCATCATGAAC R: TCATCCATCTTGTCCACCAC	#69	NM_000937
*PPIA*	F: TTCATCTGCACTGCCAAGAC R: CACTTTGCCAAACACCACAT	#158	NM_021130
*GAPDH*	F: TGGTATCGTGGAAGGACTCA R: GCAGGGATGATGTTCTGGAG	#158	NM_002046

**Table 2:** Correlations between PARP1 expression and clinical parameters of cutaneous melanoma patients.

Clinical Parameters	Parp1 Expression		
	Low (H-Score ≤ 280) (*n* = 98)	High (H-Score > 280) (*n* = 30)	*p* Value
**Age (18–86 years) ^a^**	61 (50–73)	69 (58–74)	0.090
**Gender ^b^**			0.41
Female	52 (53%)	13 (43%)	
Male	46 (47%)	17 (57%)	
**Primary tumor location ^c^**			**0.015**
Head/neck	4 (4%)	7 (23%)	
Extremities	44 (45%)	10 (33%)	
Trunk	46 (47%)	13 (43%)	
Hand/foot	4 (4%)	0 (0%)	
**Primary tumor (pT) ^a^**			0.16
pT1	27 (28%)	3 (10%)	
pT2	15 (15%)	5 (17%)	
pT3	27 (28%)	8 (27%)	
pT4	29 (30%)	14 (47%)	
**Sentinel lymph node biopsy status (SNLB) ^b^**			0.099
No metastases (SNLB-)	24 (54%)	10 (83%)	
Metastases present (SNLB+)	20 (46%)	2 (17%)	
**Regional lymph nodes status (pN) ^b^**			0.63
Metastases absent (pN-)	72 (73%)	24 (80%)	
Metastases present (pN+)	26 (27%)	6 (20%)	
**Distant metastases (pM) ^b^**			1.0
No metastases (pM-)	88 (90%)	27 (90%)	
Metastases present (pM+)	10 (10%)	3 (10%)	
**Recurrence ^b^**			0.083
No	67 (68%)	15 (50%)	
Yes	31 (32%)	15 (50%)	

^a^*p* of Wilcoxon two-sample test. ^b^ *p* value of Fisher’s exact test. ^c^ *p* value of chi^2^ test. Statistically significant results (*p* < 0.05) are presented in bold.

**Table 3:** Correlations between PARP1 expression and histopathological parameters of primary tumors in cutaneous melanoma patients.

Histopathological Parameters	PARP1 Expression		
	Low (H-Score ≤ 280) (*n* = 98)	High (H-Score > 280) (*n* = 30)	*p* Value
**Breslow thickness ^a^**			0.16
≤1 mm	27 (28%)	3 (10%)	
1.01–2.00 mm	15 (15%)	5 (17%)	
2.01–4.00 mm	27 (28%)	8 (27%)	
>4 mm	29 (30%)	14 (47%)	
**Clark level ^a^**			0.37
I	0 (0%)	0 (0%)	
II	32 (33%)	5 (17%)	
III	30 (31%)	12 (40%)	
IV	26 (27%)	10 (33%)	
V	10 (10%)	3 (10%)	
**Histological type ^b^**			0.30
Superficial spreading melanoma	47 (48%)	11 (37%)	
Nodular melanoma	47 (48%)	19 (63%)	
Acral lentiginous melanoma	4 (4%)	0 (0%)	
**Mitotic rate ^a^**			**0.0010**
0	33 (34%)	1 (4%)	
1–3	19 (20%)	7 (23%)	
≥4	45 (46%)	22 (73%)	
**Ulceration ^c^**			**0.036**
No	64 (65%)	13 (43%)	
Yes	34 (35%)	17 (57%)	
**Lymphangioinvasion ^c^**			1.0
No	92 (94%)	29 (97%)	
Yes	6 (6%)	1 (3%)	
**Tumor-infiltrating lymphocytes ^c^**			0.60
No	6 (6%)	1 (3%)	
Non-brisk	58 (59%)	21 (70%)	
Brisk	34 (35%)	8 (27%)	
**Microsatellitosis ^c^**			0.084
No	96 (98%)	27 (90%)	
Yes	2 (2%)	3 (10%)	
**Regression ^c^**			0.14
No	95 (97%)	27 (93%)	
Yes	3 (3%)	2 (7%)	

^a^*p* of Wilcoxon two-sample test. ^b^ *p* value of Fisher’s exact test. ^c^ *p* value of chi^2^ test. Statistically significant results (*p* < 0.05) are presented in bold.

**Table 4:** Correlations between PARP1 expression and clinical parameters of cutaneous melanoma patients without lymph node metastases.

Clinical Parameters	PARP1 Expression		
	Low (H-Score ≤ 280) (*n* = 72)	High (H-Score > 280) (*n* = 24)	*p* Value
**Age (24–86) ^a^**	61 (50–73)	70 (60–75)	0.055
**Gender ^b^**			1.0
Female	37 (51%)	12 (50%)	
Male	35 (49%)	12 (50%)	
**Primary tumor location ^c^**			**0.016**
Head/neck	3 (4%)	6 (25%)	
Extremities	33 (46%)	8 (33%)	
Trunk	36 (50%)	10 (42%)	
Hand/foot	0 (0%)	0 (0%)	
**Primary tumor (pT) ^a^**			**0.0030**
pT1	26 (36%)	2 (8%)	
pT2	15 (21%)	5 (21%)	
pT3	20 (28%)	5 (21%)	
pT4	11 (15%)	12 (50%)	
**Distant metastases (pM) ^b^**			1.0
No metastases (pM-)	68 (94%)	23 (96%)	
Metastases present (pM+)	4 (6%)	1 (4%)	
**Recurrence ^b^**			**0.0061**
No	60 (87%)	13 (54%)	
Yes	12 (17%)	11 (46%)	

^a^*p* of Wilcoxon two-sample test. ^b^ *p* value of Fisher’s exact test. ^c^ *p* value of chi^2^ test. Statistically significant results (*p* < 0.05) are presented in bold.

**Table 5:** Correlations between PARP1 expression and histopathological parameters of primary tumors in cutaneous melanoma patients without lymph node metastases.

Histopathological Parameters	PARP1 Expression		
	Low (H-Score ≤ 280) (*n* = 72)	High (H-Score > 280) (*n* = 24)	*p* Value
**Breslow thickness ^a^**			**0.0030**
≤1 mm	26 (36%)	2 (8%)	
1.01–2.00 mm	15 (21%)	5 (21%)	
2.01–4.00 mm	20 (28%)	5 (21%)	
>4 mm	11 (15%)	12 (50%)	
**Clark level ^a^**			0.13
I	0 (0%)	0 (0%)	
II	30 (42%)	4 (17%)	
III	22 (31%)	10 (42%)	
IV	16 (22%)	8 (33%)	
V	4 (6%)	2 (8%)	
**Histological type ^b^**			0.16
Superficial spreading melanoma	43 (60%)	10 (42%)	
Nodular melanoma	29 (40%)	14 (58%)	
Acral lentiginous melanoma	0 (0%)	0 (0%)	
**Mitotic rate ^a^**			**0.00071**
0	30 (42%)	1 (4%)	
1–3	17 (24%)	7 (29%)	
≥4	25 (35%)	16 (67%)	
**Ulceration ^c^**			**0.023**
No	53 (74%)	11 (46%)	
Yes	19 (26%)	13 (54%)	
**Lymphangioinvasion ^c^**			1.0
No	70 (97%)	24 (100%)	
Yes	2 (3%)	0 (0%)	
**Tumor-infiltrating lymphocytes ^c^**			0.26
No	4 (6%)	1 (4%)	
Non-brisk	37 (51%)	17 (71%)	
Brisk	31 (43%)	6 (25%)	
**Microsatellitosis ^c^**			1.0
No	72 (100%)	24 (100%)	
Yes	0 (0%)	0 (0%)	
**Regression ^c^**			0.15
No	71 (99%)	22 (96%)	
Yes	1 (1%)	1 (4%)	

^a^*p* of Wilcoxon two-sample test. ^b^ *p* value of Fisher’s exact test. ^c^ *p* value of chi^2^ test. Statistically significant results (*p* < 0.05) are presented in bold.

## Data Availability

The data presented in this study are available on request from the corresponding author.

## Supplementary Materials

The following are available online at https://www.mdpi.com/2073-4409/10/2/286/s1, Figure S1, Expression of PARP1 at protein level. Full immunoblots showing PARP1 and GAPDH level in cellular extracts of primary normal melanocytes (HEMn-LP and HEMn-DP) and melanoma cell lines derived from the primary tumor (A375 and WM1341D) and lymph node metastases (Hs294T and WM9) are presented. Three biological repetitions (BR) consisting of two-three technical replicates (TR) per BR were performed. Figure S2, Specificity of PARP1 antibody. Full immunoblot showing specific band of PARP1 protein (113 kDa) in cellular extracts of primary normal melanocytes (HEMn-LP and HEMn-DP) and melanoma cell lines derived from the primary tumor (A375 and WM1341D) and lymph node metastases (Hs294T and WM9) are presented. Table S1. Correlations between PARP1 expression and clinicopathologic parameters of cutaneous melanoma patients with lymph node metastases.
