# Supplementary material for: PARP1 as a Marker of an Aggressive Clinical Phenotype in Cutaneous Melanoma—A Clinical and an In Vitro Study
Source: Cells. 2021 Jan 31;10(2):286. doi: 10.3390/cells10020286 (PMC7911865; doi:10.3390/cells10020286)
Supplement: Supplementary file 1 [file cells-10-00286-s001.pdf]

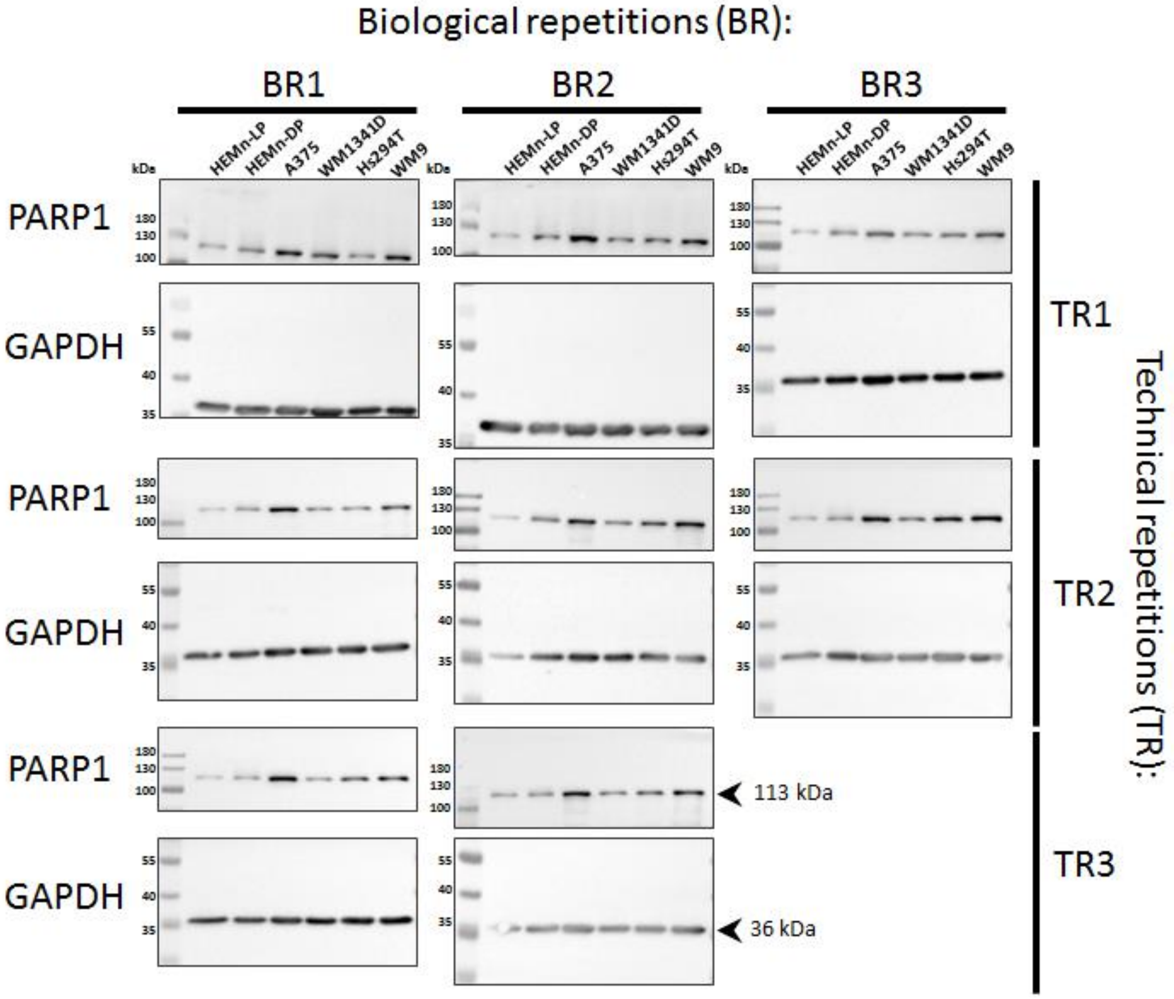

Supplementary Figure S1. Expression of PARP1 at protein level. Full immunoblots showing PARP1 and GAPDH level in cellular extracts of primary normal melanocytes (HEMn-LP and HEMn-DP) and melanoma cell lines derived from the primary tumor (A375 and WM1341D) and lymph node metastases (Hs294T and WM9) are presented. Three biological repetitions (BR) consisting of two-three technical replicates (TR) per BR were performed.

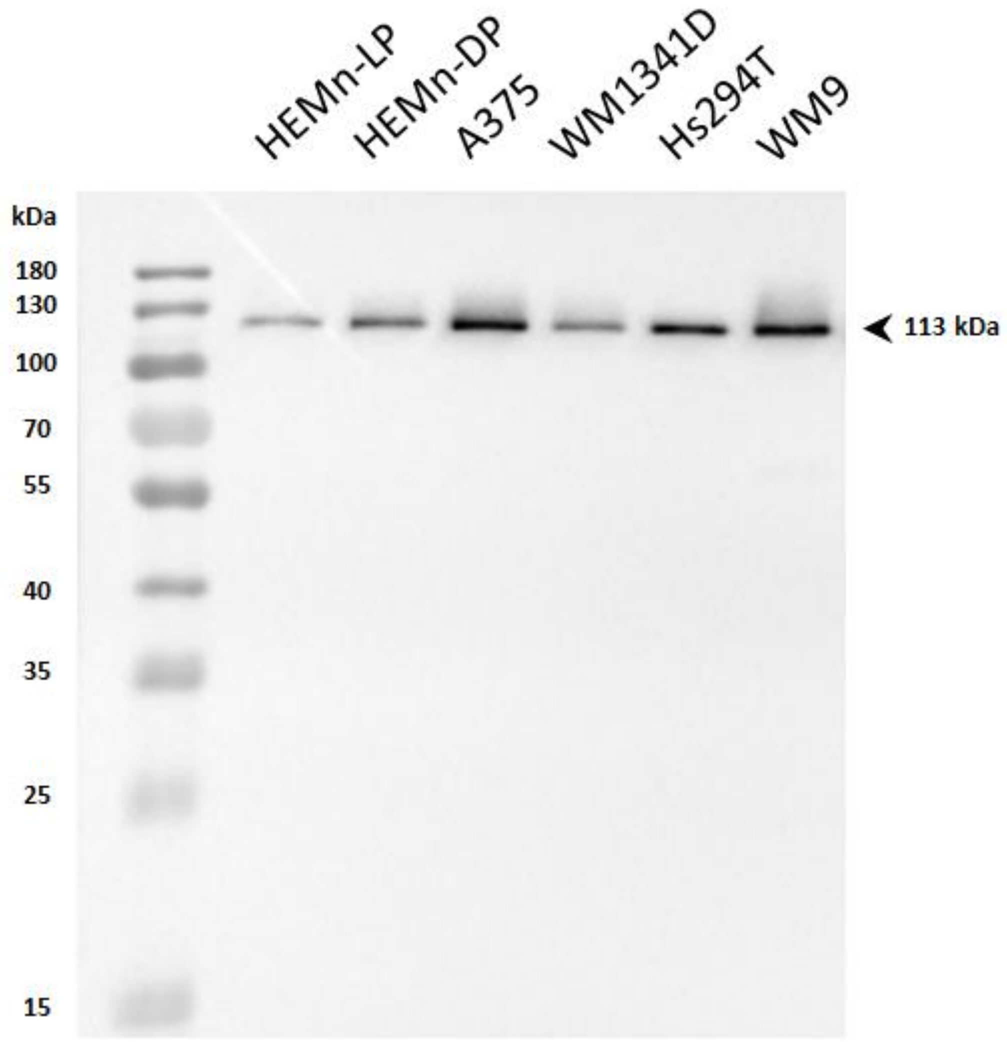

Supplementary Figure S2. Specificity of PARP1 antibody. Full immunoblot showing specific band of PARP1 protein (113 kDa) in cellular extracts of primary normal melanocytes (HEMn-LP and HEMn-DP) and melanoma cell lines derived from the primary tumor (A375 and WM1341D) and lymph node metastases (Hs294T and WM9) are presented.

Supplementary Table S1. Correlations between PARP1 expression and clinicopathologic parameters of cutaneous melanoma patients with lymph node metastases.

CLINICOPATHOLOGICAL PARAMETERS	PARP1 expression		
	Low (H-score ≤280) (n=26)	High (H-score >280) (n=6)	*p* value
**Age (18-80)** [a]	60 (53 - 73)	66 (44 - 70)	0.87
**Gender** [b]			0.17
Female	15 (58%)	1 (17%)	
Male	11 (42%)	5 (83%)	
**Primary tumor location** [c]			0.54
Head/neck	1 (4%)	1 (17%)	
Extremities	11 (42%)	2 (33%)	
Trunk	10 (38%)	3 (50%)	
Hand/foot	4 (15%)	0 (0%)	
**Primary tumor (pT)** [a]			0.22
pT1	1 (4%)	1 (17%)	
pT2	0 (0%)	0 (0%)	
pT3	7 (27%)	3 (50%)	
pT4	18 (69%)	2 (33%)	
**Distant metastases (pM)** [b]			0.62
No metastases (pM-)	20 (77%)	4 (67%)	
Metastases present (pM+)	6 (23%)	2 (33%)	
**Recurrence** [b]			1.00
No	7 (27%)	2 (33%)	
Yes	19 (73%)	4 (67%)	
**Breslow thickness** [a]			0.22
≤1 mm	1 (4%)	1 (17%)	
1.01-2.00 mm	0 (0%)	0 (0%)	
2.01-4.00 mm	7 (27%)	3 (50%)	
>4 mm	18 (69%)	2 (33%)	
**Clark level** [a]			0.92
I	0 (0%)	0 (0%)	
II	2 (8%)	1 (17%)	
III	8 (31%)	2 (33%)	
IV	10 (38%)	2 (33%)	
V	6 (23%)	1 (17%)	

**Histopathological type** [b]			0.80
Superficial spreading melanoma	4 (15%)	1 (17%)	
Nodular melanoma	18 (70%)	5 (83%)	
Acral lentiginous melanoma	4 (15%)	0 (0%)	
**Mitotic rate** [a]			1.00
0	3 (12%)	0 (0%)	
1-3	2 (8%)	0 (0%)	
≥4	20 (80%)	6 (100%)	
**Ulceration** [c]			1.00
No	11 (42%)	2 (33%)	
Yes	15 (58%)	4 (67%)	
**Lymphangioinvasion** [c]	22 (85%)	5 (83%)	1.00
No	4 (15%)	1 (17%)	
Yes			
**Tumor-infiltrating lymphocytes (TILs)** [c]			0.51
No	2 (8%)	0 (0%)	
Non-brisk	21 (81%)	4 (67%)	
Brisk	3 (12%)	2 (33%)	
**Microsatellitosis** [c]			**0.034**
No	24 (92%)	3 (50%)	
Yes	2 (8%)	3 (50%)	
**Regression** [c]			0.48
No	24 (92%)	5 (83%)	
Yes	2 (8%)	1 (17%)	

[a] *p* Wilcoxon two sample test

[b] *p* value of Fisher's exact test

[c] *p* value of chi^2 test

Statistically significant results (p<0.05)
